# GHSR agonism increases blood glucose but delays food intake in GHSR hyperresponsive rats

**DOI:** 10.1111/jne.70143

**Published:** 2026-02-13

**Authors:** Philippe Zizzari, Olalla Ramírez‐Penas, Véronique Pons, Matilda Oujezdska, Ange‐Louis Sammarcelli, Thomas Saint‐Yves, Inès Chevallier‐Chantepie, Lucile Baussart, Vanessa Nhan, Adèle Phalip, Céline Gales, Daniela Cota, Florence Noble, Jacques Pantel

**Affiliations:** ^1^ Université de Bordeaux, INSERM, Neurocentre Magendie Bordeaux France; ^2^ Université Paris Cité, INSERM, CNRS, HealthFex Paris France; ^3^ INSERM, UMR 1297, Institut des Maladies Métaboliques et Cardiovasculaires, Université de Toulouse Toulouse France

**Keywords:** ghrelin, GHSR responsivity, GPCR, glucose counter regulation, food intake, locomotion

## Abstract

Severe calorie restriction in mouse models has highlighted the crucial role of the ghrelin system in maintaining glycemia and promoting survival. We hypothesized that if ghrelin acts as a survival signal, enhancing the responsivity of the *GH secretagogue receptor* (GHSR) should favor GHSR protective responses. To test this, we used rats with genetically enhanced GHSR sensitivity (*Ghsr* ^Q343X^) and wild‐type littermate controls and examined their acute responses to pharmacological challenges. Consistent with our hypothesis, *Ghsr* ^Q343X^ rats, despite normal glucose and insulin tolerance, exhibited a significant increase in blood glucose in response to GHSR agonism, accompanied by elevated counter‐regulatory hormones including corticosterone. Concurrently, these rats displayed a notable decrease in locomotor activity and delayed feeding response. Also, GHSR agonism partially altered the cocaine‐induced hyperlocomotion of *Ghsr* ^Q343X^ rats while they showed unaltered locomotor sensitization to cocaine. At the cellular level, functional studies indicated that the *Ghsr* ^Q343X^ mutation prolongs ghrelin‐induced GHSR‐G protein canonical signaling. Altogether, in a model of increased GHSR sensitivity, GHSR agonist stimulation was sufficient to promote a robust blood glucose increase, while the acute feeding response was delayed in a context of unexpected hypolocomotor response. This mechanism may have implications for severe states of undernutrition such as restrictive anorexia nervosa.

## Graphical abstract

GHSR agonism increases blood glucose, inhibits locomotor activity and delays food intake in GHSR hyperresponsive rats.

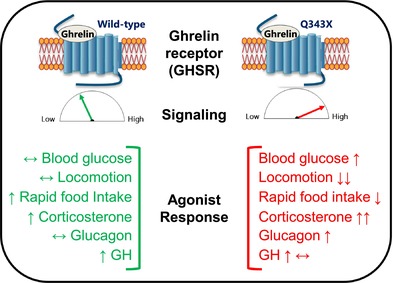

## 1 INTRODUCTION

The gut hormone ghrelin is a pleiotropic hormone involved in growth hormone (GH) secretion, glucose and energy homeostasis, as well as behavior, including food seeking, reward and memory.^1^ Current concepts support that nutritional status significantly influences the biological properties of ghrelin.^2^ Seminal studies highlight the key protective role of the ghrelin system against glucopenia in genetic mouse models under life‐threatening conditions of chronic severe caloric restriction. In these conditions, mice with genetic invalidation of *Ghrl*, *Ghsr* or *Goat* genes,^3^, ^4^, ^5^ the partial *Ghsr* loss‐of‐function mutation *Ghsr* ^A203E^ ^6^ or the toxic ablation of ghrelin cells in adults^7^ exhibit an exaggerated drop in blood glucose. Conversely, under the fed conditions, the physiological effects of this hormone are minimal as indicated by numerous genetic studies.^8^ In conditions of energy excess, obesity is characterized as a state of central ghrelin resistance.^9^, ^10^

The relationship between ghrelin and the system known to oppose hypoglycemia, referred to as glucose‐counter regulatory response (CRR), was recently examined in models of insulin‐induced hypoglycemia. Mice with genetic invalidation of the *Ghrl* gene (*Ghrl* ^*−/−*^ *mice*) compared to wild‐type mice show a more pronounced hypoglycemia in response to acute insulin requiring a higher glucose infusion rate and less robust corticosterone and GH responses during hyperinsulinemic–hypoglycemic clamps, which can be countered by GHSR agonism.^11^ Similarly, in a model of type 1 diabetes, streptozotocin‐treated *Ghrl* ^*−/−*^ mice also required a higher glucose infusion rate and attenuated epinephrine and norepinephrine response compared to streptozotocin‐treated wild‐type mice.^12^ These findings reveal that endogenous ghrelin is permissive for the CRR process. Collectively, these models, all involving loss‐of‐function in the ghrelin system, underscore to varying degrees a global role for ghrelin during severe nutritional stress, aligning with a survival role for this hormone.^2^

A single G protein‐coupled receptor (GPCR), known as the *GH secretagogue receptor* (GHSR), mediates ghrelin's key properties.^13^ The physiological importance of GHSR signaling is underscored by its elaborate regulation.^14^ Beyond GHSR signaling related to ghrelin agonism, the GHSR exhibits constitutive activity,^6^, ^15^, ^16^ GPCR heterodimer regulation,^17^ and GHSR antagonism/inverse‐agonism by the *liver‐expressed antimicrobial peptide 2* (LEAP2), a hormone showing opposite regulation to ghrelin by energy stores.^18^, ^19^ According to this latter mechanism, situations characterized by enhanced LEAP2 over ghrelin levels are predicted to dampen the effects of ghrelin (i.e., fed state or obesity), while negative energy balance conditions are relatively permissive to ghrelin's effects.^20^ In summary, this unique mechanism for an endocrine system places the sensitivity of the GHSR for ghrelin at the heart of this system's physiology.

Altogether, if blood ghrelin acts as a survival signal, enhancing GHSR responsivity should favor ghrelin's protective effects. To test this hypothesis, we used rats with genetically enhanced GHSR sensitivity. These rats have the *Ghsr* ^Q343X^ mutation that predicts the deletion of a 22 amino acid domain involved in the termination of GHSR‐G protein signaling.^21^ Transfected cells expressing the mutated isoform showed impaired ghrelin‐induced internalization and ghrelin‐induced β‐arrestin recruitment compared to control cells. Additionally, homozygous rats expressing the *Ghsr* ^Q343X^ allele (M/M rats) displayed increased GH release and 4‐h chow intake at low doses of ghrelin/GHSR agonist. Furthermore, M/M rats developed fat accumulation and insulin resistance with age compared to wild‐type littermate controls (WT/WT).

In this study, we compared *Ghsr* ^Q343X^ mutant rats with WT/WT rats exploring their acute responses to pharmacological and metabolic challenges on blood glucose and feeding behavior. Our results reveal a remarkable pharmacological response pattern to GHSR agonism in *Ghsr* ^Q343X^ rats, which may recapitulate the ghrelin response in survival conditions.

## 2 MATERIALS AND METHODS

### 2.1 Animals

The mutant line of rats carrying the *Ghsr* ^Q343X^ allele, initially obtained from the Medical College of Wisconsin (Milwaukee, WI, USA) in rats of the *fawn hooded hypertensive* (FHH) genetic background (FHH‐Ghsrm1Mcwi rats),^21^ was backcrossed in our animal core facility (BioMedTech, Université Paris Cité) over 10 generations with Wistar rats obtained commercially (Janvier Labs, France). Homozygous rats for the *Ghsr* ^Q343X^ allele and wild‐type littermates are referred to as M/M and WT/WT rats, respectively. Rats used in this study (445 rats from 13 litters) were obtained by heterozygous breeding. Animals were raised four by cages with free access to water and chow diet (A04, SAFE), in a room with controlled temperature (22–24°C) and illumination (12‐h light:12‐h darkness schedule with lights on at 7:00 am). The body weight of rats was collected at least at 3 and 8 weeks‐old for ANCOVA analyses of weight gain. During experiments, rats remained in their home cage in groups unless indicated. The genotype of the rats at the *Ghsr* locus was determined on genomic DNA extracted from ear punch using the Sensifast HRM kit (Meridian Bioscience) and the following couple of primers (CCTGGTGTCCTTTGTCCTCT and GCTTCGGGTGTAGAGCAATG). Experiments were performed with male and female adult (8–11 weeks old) or adolescent rats with ad libitum access to food and water, unless otherwise specified.

### 2.2 Glycemia measurements

Tail blood glucose concentrations were measured using a handheld glucometer (OneTouch verio reflect™) before (*t*_0_) and at several timings after injection of the GHSR agonist macimorelin (AEZS130, Aeterna Zentaris) or rat ghrelin (GenScript) as indicated in each figure legend. To standardize blood sampling, food access was removed at 10 a.m. in all experiments involving ad libitum fed rats and glycemia measures were initiated at 1 p.m. Rats involved in the 4‐days calorie restriction were fed 40% of the regular chow diet in their grouped cages at dark onset.

### 2.3 Glucose and insulin tolerance tests

For the analysis of glucose tolerance in rats pretreated with saline or the GHSR agonist AEZS130 (1E^−6^ mol/kg, s.c.), 18 h‐fasted rats were i.p. injected with 2 g/kg of D‐glucose (Sigma‐Aldrich). Tail blood glucose concentrations were measured before (*t*_0_) and at 15, 30, 60, and 120 min after injections. For the determination of insulin sensitivity of rats, food was removed 3 h before i.p. injection with 1.0 UI/kg of insulin (Umuline, Lilly) performed at 1:00 p.m. Tail blood glucose concentrations were measured before (*t*_0_) and at 10, 20, 30, 60, 90, 120, 150, and 180 min after injection. The glucose disappearance rate (KITT) was calculated as the slope of the decreasing line of blood glucose levels over 20 min from insulin administration.

### 2.4 Food intake assays

These assays were performed in individual locomotor cages (Imetronic). The locomotor activity was measured as beam‐break counts. The 30‐min chow intake assay involved 4 sessions per rat involving a habituation session (with no injection and no food), and 3 successive injection sessions with food access (saline, GHSR agonist AEZS130 at 1E^−6^ mol/kg and 3E^−6^ mol/kg, s.c.). Sessions occurred every 2–3 days. Each rat received its injection at a fixed hour in the morning just before entering the cage, and the food rack containing pre‐weighed food (chow) was added 10 min after the injection. The 30‐min chow intake was calculated by subtraction. In the 4‐h chow intake assay, each rat was subjected to 6 sessions beginning at 10:00 a.m., every 2–3 days, all in the presence of pre‐weighed food in the cage. The two first sessions successively involved a habituation session (without injection) and a saline injection. Then, sessions were repeated in a crossover‐designed manner so that the animals were randomly assigned to receive saline or AEZS130 s.c injection (1E^−8^, 1E^−7^, 1E^−6^ mol/kg). The chow intake of each rat was calculated by subtraction, weighting the remaining pellets at two timings (hour 1 and hour 4).

### 2.5 Hormone measurements

Fed rats were habituated to manipulation and to a saline injection. During the protocol, performed in the absence of food access, each rat received 2 s.c. injections spaced 2–7 days apart. AEZS130 (3E^−6^ mol/kg) and saline injections were injected between 9:30 and 10:30 a.m., according to a random crossover design protocol. Blood sampling in live animals was performed from a tail nick using two heparinized capillaries 15‐min post‐injection. Blood samples maintained at 4°C were centrifuged and plasma was stored at −80°C until measurements. Plasma growth hormone concentrations were determined by a double‐antibody enzyme immunoassay using materials supplied by the National Hormone and Peptide Program as previously described^21^ and a GH standard (Sigma). The other hormone concentrations were determined by commercially available ELISA kits: insulin (Mercodia), glucagon (Mercodia), and corticosterone (Arbor assays).

### 2.6 Cell culture and transfection

HEK293T/17 cells (human embryonic kidney) were maintained in DMEM AQmedia (Sigma. St. Louis, MO) supplemented with 10% fetal bovine serum (Life technologies) and 100 units/mL penicillin and 100 μg/mL streptomycin at 37°C in a humidified atmosphere containing 5% CO_2_. Twenty‐four hours after cell splitting, transient transfections were performed using polyethylenimine (PEI, Polysciences Inc.).

### 2.7 Bioluminescence resonance energy transfer (BRET) measurements

G protein activation was performed as previously described.^22^ Briefly, vectors encoding receptors, Gα‐RLuc8, GFP2‐Gγ2, and Gβ1 were transiently cotransfected into HEK293T/17 cells as indicated in the figure legends. Forty‐eight hours after transfection, cells were washed, resuspended in PBS containing 0.1% (w/v) glucose at room temperature, and then distributed (80 μg proteins/well) in a 96‐well microplate (PerkinElmer). Cells were incubated in the absence (basal BRET) or in the presence of ghrelin for different time periods as indicated in the figure legends. BRET signal between RLuc8 and GFP2 was measured after the addition of the luciferase substrate coelenterazine 400a (5 μM). BRET^2^ readings were collected using a modified Infinite F500 (Tecan Group Ltd), and the BRET^2^ signal was calculated by the ratio of emission of GFP2 (510–540 nm) to RLuc8 (370–450 nm).

### 2.8 Data and statistical analyses

Results are presented as mean ± SEM with sample sizes (n) and P values detailed in the figure captions. In the glucagon assay, values below the detection limit (DL) were substituted with DL/√2 to handle undetermined measurements. All statistical analyses were conducted using GraphPad Prism® 8.0.2 software. Differences between two groups were assessed using non‐parametric Mann–Whitney or Wilcoxon tests, or parametric Student's *t*‐tests, as appropriate. For comparisons involving multiple groups, two‐way ANOVA or repeated measure ANOVA were employed, with *p* value of post hoc tests adjusted using the Sidak correction. Alternatively, in insulin and corticosterone determinations, few missing values led to the use of a mixed‐effects model. In the case of blood glucose measures of the experiment with feeding conditions fluctuations, three‐way repeated measure ANOVA were used. Comparisons of body weight gain from 3 to 8 weeks of age were analyzed using linear mixed‐effects models (lmer function, *lme4* R package) to account for the hierarchical structure of the data (rats nested within litters). Two models were compared: a basic model including random intercepts for Litter and ID (Weight ~ Time * Genotype * Sex + (1 | Litter) + (1 | ID)), and an improved model adding random slopes for Time within Litter (Weight ~ Time * Genotype * Sex + (Time | Litter) + (1 | ID)). The improved model was selected based on a significantly better fit (ΔAIC = 55.8, ΔBIC = 46.2; *χ* ^2^(2) = 58.6, *p* < .001). Model assumptions were verified by inspecting residuals for homoscedasticity and normality.

### 2.9 Study approval

The procedures involving rats were approved by the local ethics committee on animal experimentation (CEEA‐040).

## 3 RESULTS

### 3.1 GHSR agonism dose‐dependently increases blood glucose in M/M rats regardless of nutritional status

**FIGURE 1:**
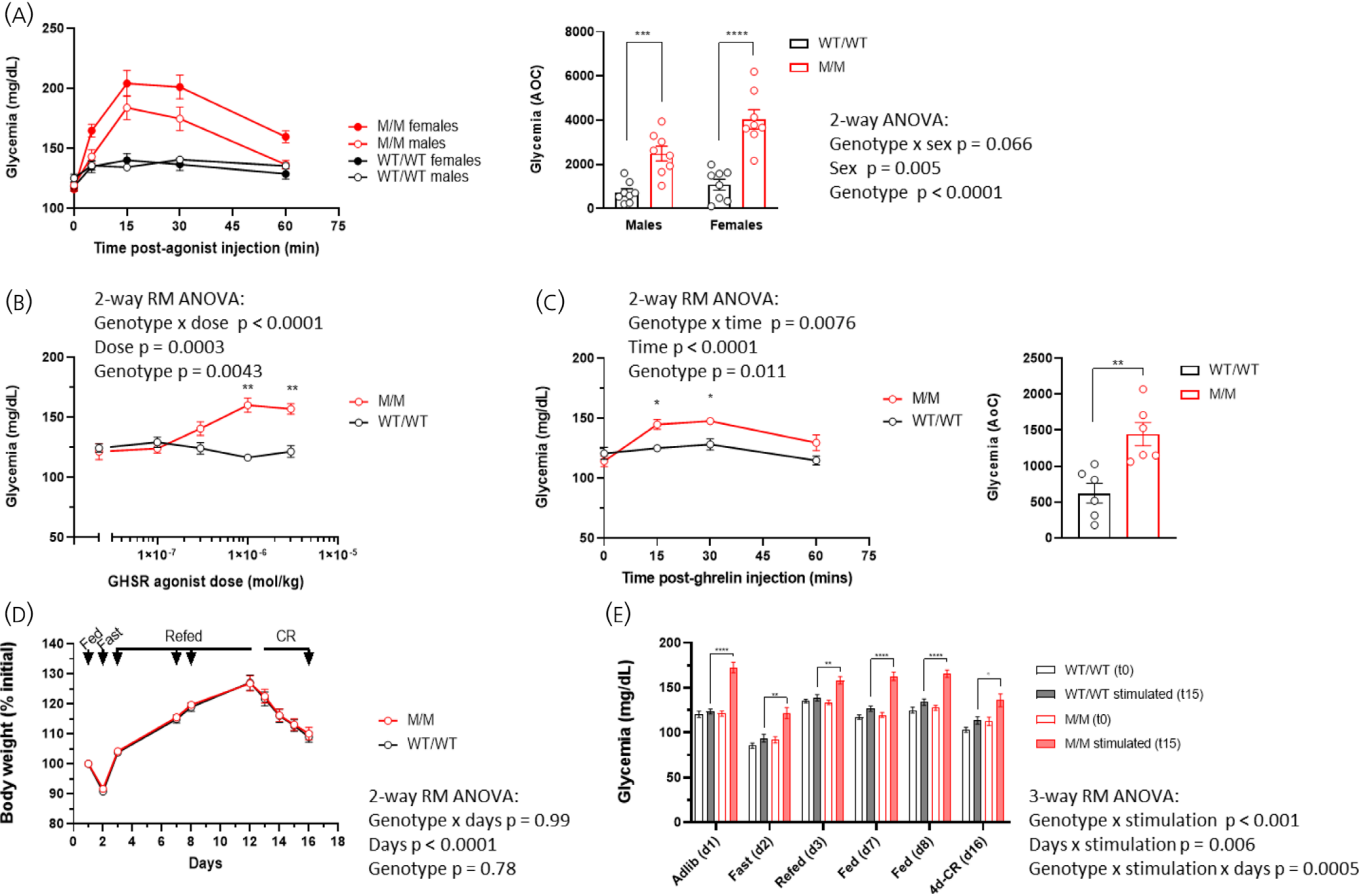
M/M rats exhibit a robust blood glucose increase to GHSR agonism regardless of nutritional status. Glycemic response over time in adult M/M and WT/WT rats (*n* = 8 per sex and per genotype) injected with the GHSR agonist (AEZS130, 1E^−6^ mol/kg, s.c.) (left panel) and the total response calculated as the area of the curve (right panel) (A). Glycemic response at 15 min after the s.c. injection of increasing doses of the GHSR agonist (AEZS130, s.c.) in a subgroup of male rats (*n* = 6 per group) (B). Glycemic response over time in a group of adolescent M/M and WT/WT male rats (*n* = 6 per genotype) injected with ghrelin (1E^−7^ mol/kg, s.c.) (left panel) and the total response calculated as the area of the curve (AoC) (right panel) (C). Fluctuations in body weight and glycemia before and after the injection of a GHSR agonist in adolescent rats (*n* = 6 per genotype and per sex) subjected to a 16‐day protocol including fasting (24 h), refeeding (10 days), and calorie restriction (4 days) episodes (D, E). Arrows indicate days with glycemia measurements. Unless otherwise specified, experiments include female and male rats in similar proportions. Data are presented as mean ± SEM and were analyzed using 2‐way or 3‐way ANOVA and post hoc tests where relevant, or Student's *t*‐test when specified. ^≈^ *p* < .1, *p < .05, **p < .01, ***p < .001, ****p < .0001 (Sidak's post hoc test). All specific statistical information is reported in Table S1.

Given the protective role of the ghrelin system in glucopenia, we investigated whether rats with genetically enhanced GHSR sensitivity might exhibit an elevated glycemic response to GHSR stimulation. To this end, we stimulated homozygous rats carrying the *Ghsr* ^Q343X^ allele (M/M) and wild‐type littermate control rats (WT/WT) using a GHSR agonist injection. As shown in Figure 1A, both female and male M/M rats had a clear increase in glycemia, peaking at 15‐min post‐injection with a challenge dose of the agonist, and then returning to normal levels at the 60‐min time point. In contrast, WT/WT rats injected similarly showed globally unchanged glycemia. Furthermore, two‐way ANOVA analyses comparing M/M rats revealed a stronger total response in female as compared to male rats (*p* < .01). Figure 1B indicates that M/M and WT/WT rats injected daily with increasing doses of the GHSR agonist exhibited a genotype × dose interaction, supportive of a dose–response curve only in M/M rats. To ensure that the enhanced glycemic response of M/M rats was not specific to the GHSR full agonist used, we compared the glycemic response of M/M and WT/WT littermate controls injected peripherally with ghrelin (Figure 1C). M/M rats responded to ghrelin while control rats did not, suggesting that GHSR agonism, in general, could trigger a clear blood glucose elevation in rats carrying the *Ghsr* ^Q343X^ allele.

To assess whether the blood glucose increase in response to GHSR pharmacological stimulation could be influenced by feeding conditions, adolescent M/M and WT/WT littermate control rats were subjected to GHSR stimulation tests under fed, fasted, refed or calorie restriction conditions, as shown in Figure 1D. Body weight fluctuations were similar among genotype groups throughout the protocol. Basal unstimulated glycemia fluctuated according to the feeding conditions but did not differ between genotype groups (Figure 1E). The estimated marginal means revealed significant differences in the glycemic response to stimulation between WT/WT and M/M rats. Globally, the WT/WT rats showed a moderate but significant increase in blood glucose levels after stimulation (from 114.0 ± 2.84 mg/dL to 121.4 ± 2.84 mg/dL, Δ = +7.39 units, *t*(22) = −2.599, *p* = .0164), while M/M rats exhibited a much stronger response (from 118.0 ± 2.84 mg/dL to 152.9 ± 2.84 mg/dL, Δ = +34.96 units, *t*(22) = −12.296, *p* < .0001). By nutritional status, the response to stimulation in WT/WT rats was significant in 4d‐CR (Δ = +10.67, *p* = .0227), fed (d7, Δ = +9.50, *p* = .0419), and fed (d8, Δ = +9.00, *p* = .0536), but not in adlib (*p* = .5165), fast (*p* = .0708), or refed (*p* = .4177). In contrast, M/M rats showed a highly significant response across all nutritional statuses (all *p* < .0001), with increases ranging from +23.50 units (4d‐CR) to +51.00 units (adlib). These results indicate that M/M rats exhibit a hypersensitive response to stimulation compared to WT/WT rats, regardless of nutritional status.

### 3.2 M/M rats exhibit unaltered glucose or insulin tolerance

**FIGURE 2:**
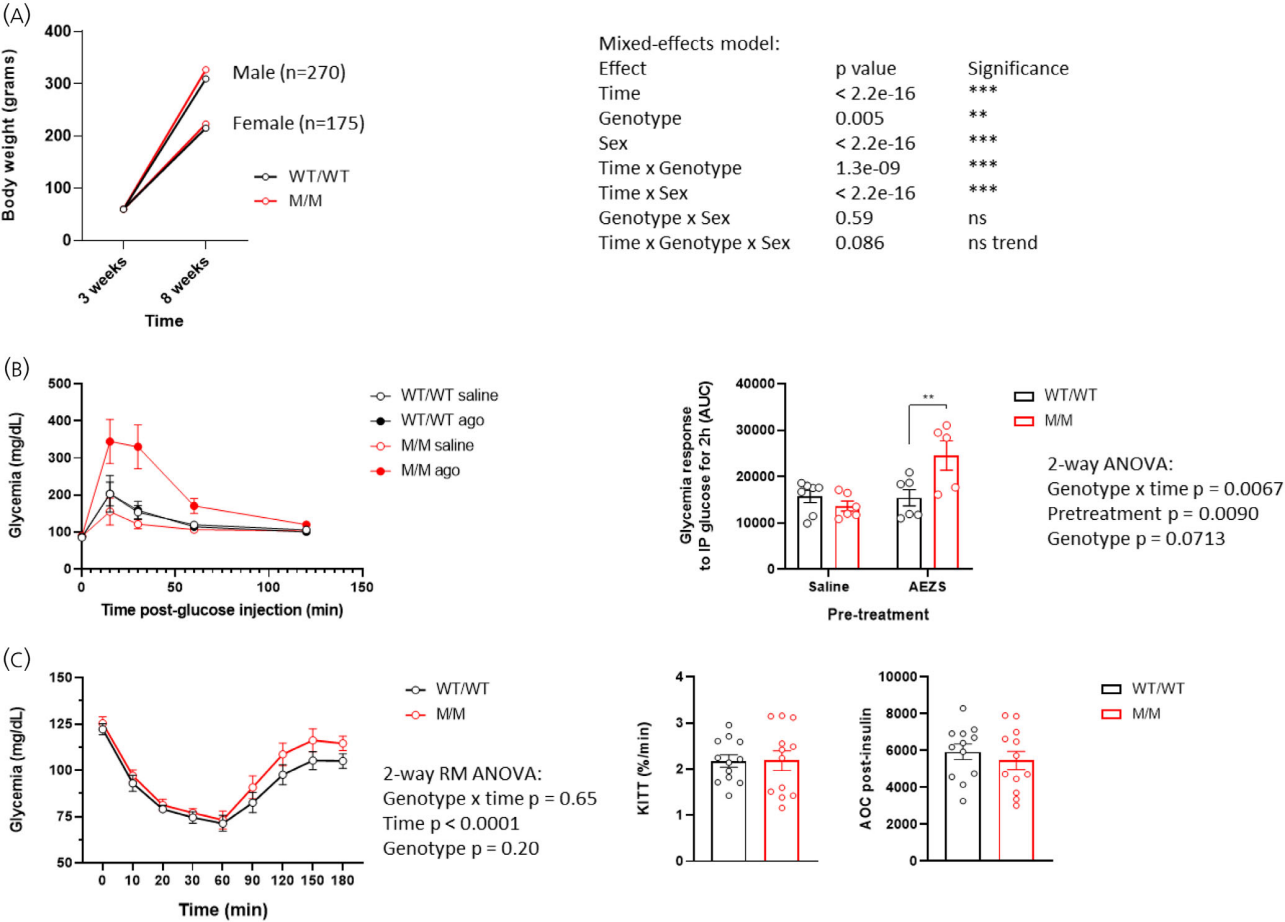
M/M rats show minimally increased body weight and unaltered glucose or insulin tolerance. Comparison of the body weight of M/M and WT/WT rats at weaning age (3 weeks old) and at 8 weeks old. Statistical analyses used a linear mixed‐effects model including random slopes for time within Litter (A). Glycemic response to an i.p. glucose tolerance test (GTT) in M/M and WT/WT male rats (*n* = 5–7 per genotype and per treatment group) pretreated with the GHSR agonist (AEZS130, 1E^−6^ mol/kg, s.c.) or saline; the time course (left panel) and the total response (right panel), calculated as the area under the curve (AoC), are shown (B). Glycemic response to an insulin tolerance test (ITT) (1.0 UI/kg, i.p.) in M/M and WT/WT rats (*n* = 12 per genotype); the time course (left panel), the plasma glucose decay rate as the KITT determination (middle panel), and the total ITT response, calculated as the area of the curve (right panel), are shown (C). Analyses of KITT or the AoC were performed with a Student *t*‐test. Unless otherwise specified, experiments include female and male rats in similar proportions. Data are presented as mean ± SEM and were analyzed using repeated‐measures 2‐way ANOVA and post hoc tests where relevant, or Student *t*‐test. **p < .01, (Sidak's post hoc test). All specific statistical information is reported in Table S1.

Based on the GHSR agonist‐induced hyperglycemia observed in M/M rats, we assessed body weight gain and glucose homeostasis in these rats compared to their WT/WT littermate controls. As shown Figure 2A, analyses of body weight from weaning to adulthood using linear mixed‐effects models revealed a modest but significant increase in weight in M/M rats compared to WT/WT rats by 6.2% in males and 2.3% in females. This suggests that genetically enhanced GHSR sensitivity favors minimal weight gain at 8 weeks of age. We then evaluated glucose and insulin tolerance of M/M rats. Figure 2B indicates that M/M rats did not differ from WT/WT control rats in an i.p. glucose‐tolerance test. However, when a GHSR agonist injection preceded the test, M/M rats showed enhanced glycemia compared to WT/WT controls, thus confirming the enhanced GHSR responsivity of M/M rats. Finally, during an insulin tolerance test, M/M rats displayed a glucose response that did not significantly differ from that of WT/WT rats, as supported by similar KITT values and the total response calculated as the area of the curve (Figure 2C). Overall, these results indicate that while M/M rats show minimal body weight gain over littermate controls during infancy, they exhibit unaltered glucose tolerance or insulin sensitivity, at least as young adults (8–10 weeks old).

### 3.3 M/M rats show decreased locomotor activity and food intake in the acute response to a GHSR agonist

**FIGURE 3:**
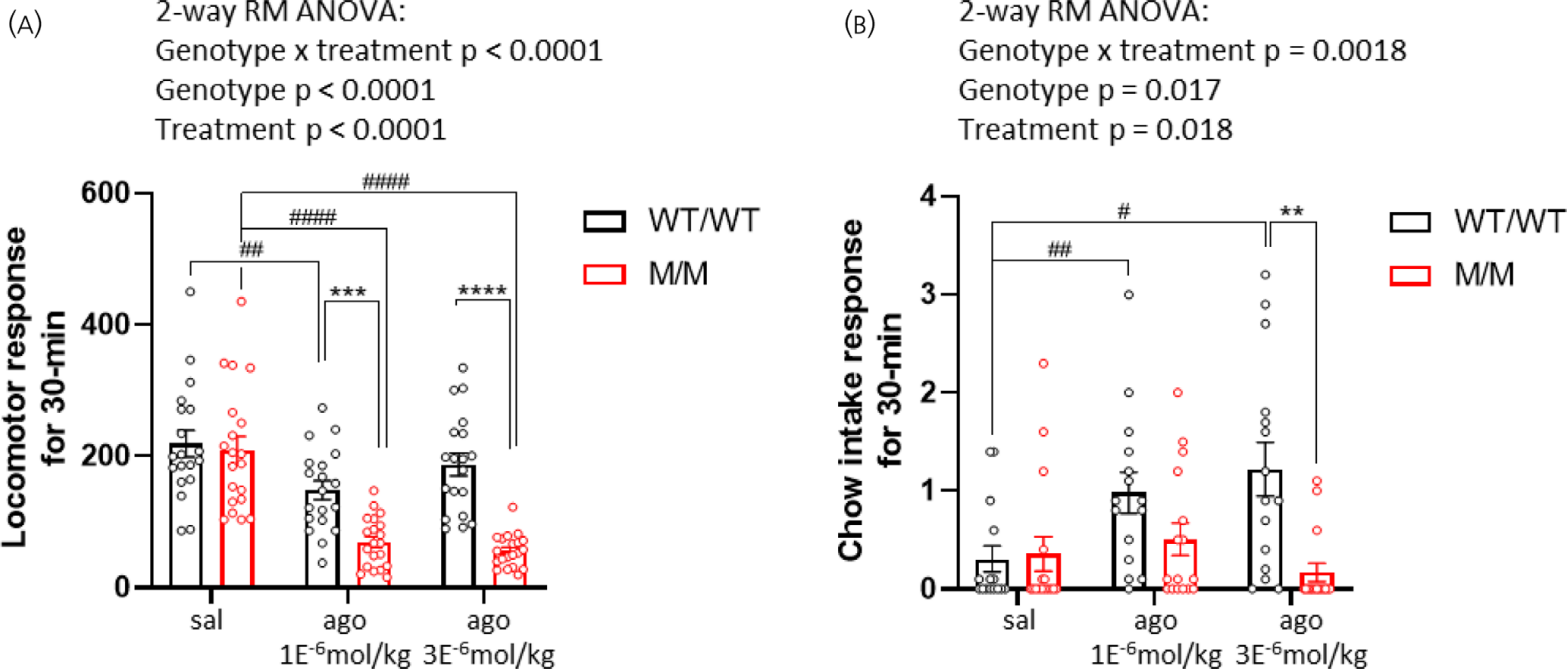
M/M rats show decreased locomotor activity and food intake in the early phase of the response to the GHSR agonist. Horizontal locomotion (A) and food intake (B) over 30 min in response to s.c. injection of the GHSR agonist (AEZS130) or saline in WT/WT and M/M rats (10 rats per genotype group and per sex) in locomotor cages. The experiment includes female and male rats with similar proportions. Data were analyzed using repeated measures 2‐way ANOVA and Sidak's post hoc tests comparing genotype groups (*) or treatments within a same genotype (#) where relevant. Data are presented as mean ± SEM. *p < .05, **p < .01, ***p < .001, ****p < .0001. All specific statistical information is reported in Table S1.

During the aforementioned agonist‐induced glycemic experiments, we observed that approximately half of the rats displayed behavioral signs of hypo‐activity and hypotonia when handled. Notably, all rats exhibiting these signs were of the M/M genotype. We therefore assessed whether GHSR agonism could alter the spontaneous behavior of M/M rats. To test this, we conducted a feeding assay in M/M and WT/WT rats injected with either saline or the GHSR agonist during a short time interval (30 min) to measure beam‐break counts and chow intake. As indicated in Figure 3A, the agonist treatment decreased the locomotion of rats, and M/M rats responded more strongly than WT/WT rats at the doses of agonist tested. Regarding the food intake response, WT/WT rats showed a potent response to the agonist over saline treatment (by 385% and 435% for doses of 1E^−6^ and 3E^−6^ mol/kg, respectively), whereas M/M rats showed no feeding response (Figure 3B). Collectively, these findings indicate that GHSR agonism in M/M rats can alter the food intake response and locomotor activity within a short timeframe post‐injection.

### 3.4 M/M rats exhibit a biphasic feeding response to a GHSR agonist

**FIGURE 4:**
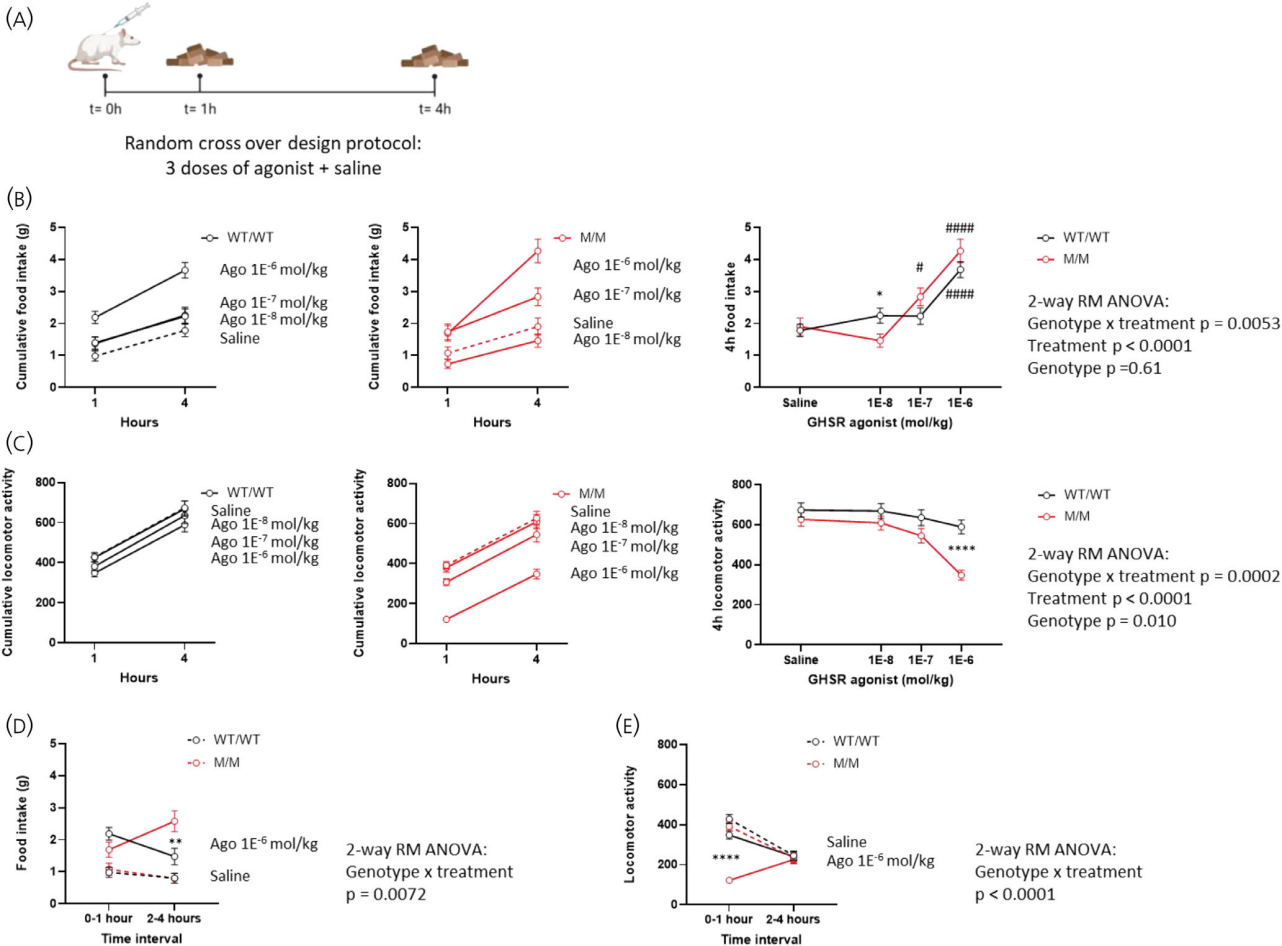
M/M rats show a biphasic feeding response to GHSR agonism. Study design: the chow intake and the locomotor response of M/M and WT/WT rats (*n* = 16 per genotype and per sex) were monitored at two time points (1st hour and 4th hour) in response to s.c. injections of saline and increasing doses of the GHSR agonist AEZS130 (1E^−8^, 1E^−7^, 1E^−6^ mol/kg) in a randomized crossover design protocol (A). Cumulative food intake as a function of time and analyses of the 4‐h food intake as a function of the treatment dose in WT/WT and M/M rats (B). Cumulative locomotor activity as a function of time and analyses of the 4‐h locomotor activity as a function of the treatment dose in WT/WT and M/M rats (C). Temporal analyses of the response of WT/WT and M/M rats injected with saline or the highest dose of agonist regarding food intake (D) and locomotor activity (E). Data were analyzed using repeated measures 2‐way ANOVA and Sidak's post hoc tests comparing genotype groups (*) or GHSR agonist to saline treatment within the same genotype (#) where relevant. Data are presented as mean ± SEM. *p < .05, **p < .01, ****p < .0001. All specific statistical information is reported in Table S1.

Given that M/M rats displayed a behavioral inhibition in response to the GHSR agonist injection—a response capable of altering the early feeding response—we examined the feeding response of rats over a longer period to identify a plausible catch‐up effect. For this purpose, we measured the increase in food intake at 1 and 4 h after injecting WT/WT and M/M rats with increasing doses of the GHSR agonist (Figure 4A). As shown in Figure 4B, analyses performed on the total 4‐h intake support a dose‐dependent increase of food intake, but no clear differences among genotypes. Still, M/M rats significantly increased their food intake over the saline control starting from the intermediate agonist dose (1E^−7^ mol/kg), unlike WT/WT rats. Analyses performed on the total locomotor activity revealed a dose‐dependent decrease of locomotor activity that differed between genotypes (Figure 4C). To distinguish the contribution of time in these pharmacological responses, we then compared feeding and locomotor responses for each time interval. These revealed an enhanced feeding response of M/M over WT/WT rats in the 2–4 h interval at the highest agonist dose (1E^−6^ mol/kg), with intakes of 316% and 189% relative to saline treatment, respectively (Figure 4D). In contrast, at this same dose of agonist, the locomotor response of M/M rats was clearly decreased during the first hour compared to WT/WT rats, but not during the 2–4 h interval (Figure 4E).

Collectively, M/M rats injected with the highest dose of the GHSR agonist exhibit enhanced pharmacological responses compared to WT/WT controls, characterized by non‐overlapping kinetics: (*i*) decreased beam‐break counts during the first hour and (*ii*) enhanced food intake during the 2–4 h interval, suggesting a competition between locomotor and feeding responses over time.

### 3.5 Psychomotor response to cocaine in M/M rats

**FIGURE 5:**
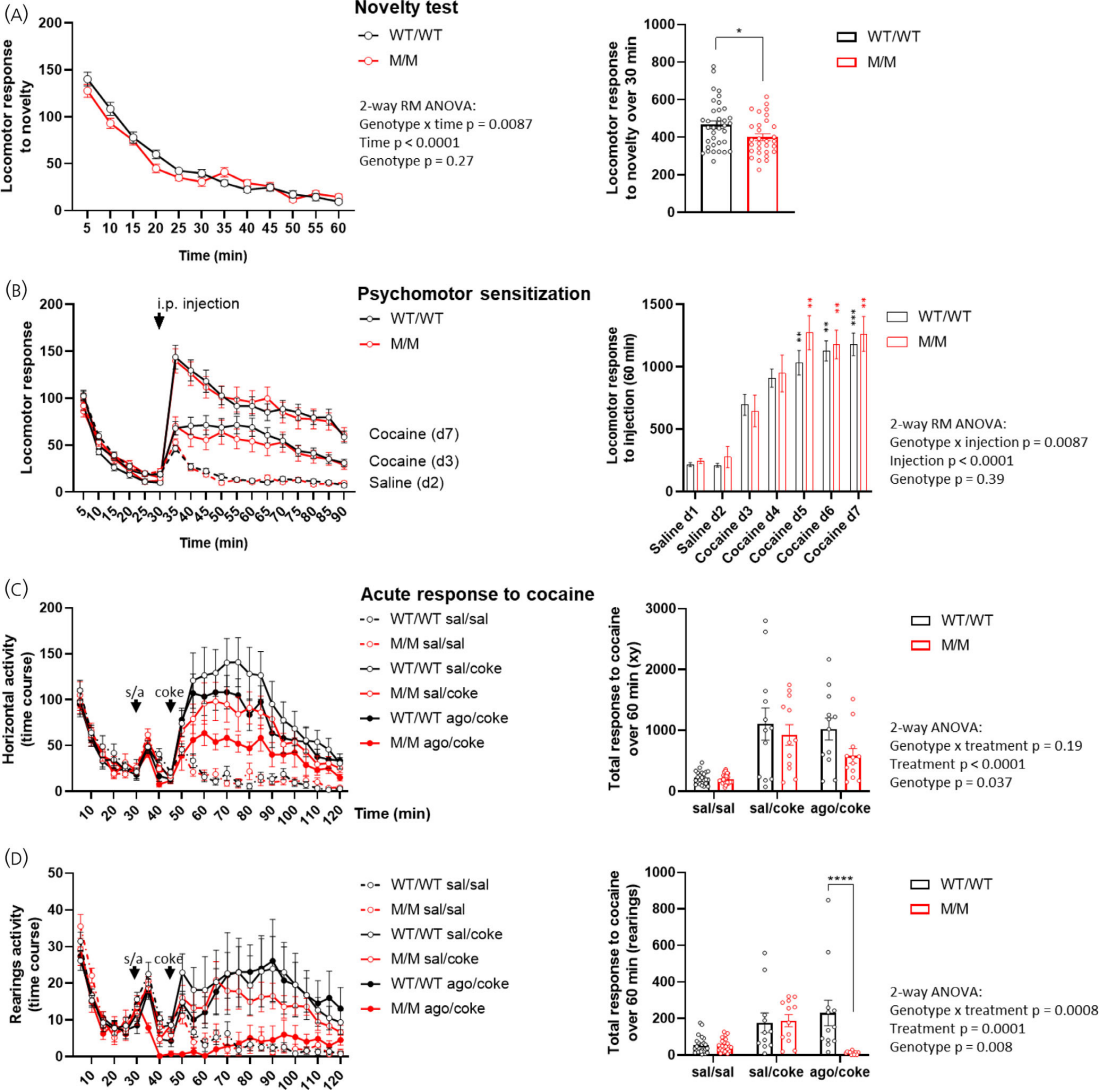
Cocaine‐induced hyperlocomotion in M/M rats. Locomotor response to novelty in male WT/WT (*n* = 36) and M/M (*n* = 30) rats: Time course and total response over 30 min (A). Cocaine‐induced psychomotor sensitization in same rats in response to saline (days 1–2) and iterative cocaine injections (20 mg/kg, i.p.) (days 3–7): Time course and the total response to the injection over 60 min (B). Effect of GHSR agonist pretreatment on cocaine‐induced hyperlocomotion in WT/WT and M/M rats (C, D). Groups of naive rats (*n* = 6 per genotype and treatment group) received saline or GHSR agonist (1E^−6^ mol/kg)) pretreatment injected s.c. at 30 min and cocaine (20 mg/kg) or saline injected i.p. at 45 min. Shown are the time course (left panel) and the 60‐min total response to cocaine (right panel) regarding horizontal activity and rearing activity (C and D, respectively). Unless otherwise specified, experiments include female and male rats with similar proportions. Shown are mean ± SEMs of beam break counts: horizontal activity (A–C) or rearings (D). Data were analyzed using 2‐way ANOVA and post hoc tests when relevant, or Student *t*‐test. *p < .05, **p < .01, ***p < .001, ****p < .0001. All specific statistical information is reported in Table S1.

The ghrelin system is recognized for its role in dopaminergic neuromodulation.^23^, ^24^ To investigate whether this relationship contributes to the previously mentioned locomotor abnormalities observed in GHSR‐stimulated M/M rats, we compared the locomotor response of M/M and WT/WT rats following cocaine treatment. First, we assessed the capacity of rats to develop psychomotor sensitization to repeated cocaine injections. Prior to the protocol, M/M rats exhibited a modest yet significant reduction in locomotor response to novelty compared to WT/WT rats (Figure 5A). However, both M/M and WT/WT rats displayed similarly increased locomotor responses to cocaine injections at equivalent doses and with similar kinetics (Figure 5B), suggesting unaltered sensitization to cocaine in M/M rats. Second, to further explore the decreased locomotion observed in GHSR‐stimulated M/M rats, we examined the impact of a GHSR agonist on acute cocaine‐induced hyperlocomotion using a new cohort of rats. As anticipated, an acute cocaine injection induced hyperlocomotion in both genotype groups compared to saline‐treated rats. Treatment with the GHSR agonist did not affect the cocaine response in WT/WT rats compared to saline‐treated controls (Figure 5C,D). Conversely, while this treatment failed to inhibit cocaine‐induced horizontal activity response in M/M rats, it successfully impaired their rearing response.

Overall, these results suggest that M/M rats retain the ability to develop behavioral sensitization to cocaine, a mechanism known to involve the GHSR‐β‐arrestin pathway.^25^ However, they may exhibit partially altered cocaine‐induced hyperlocomotion when stimulated by a GHSR agonist.

### 3.6 The acute response of M/M rats to GHSR agonism involves enhanced glucose counter‐regulatory response mediators

**FIGURE 6:**
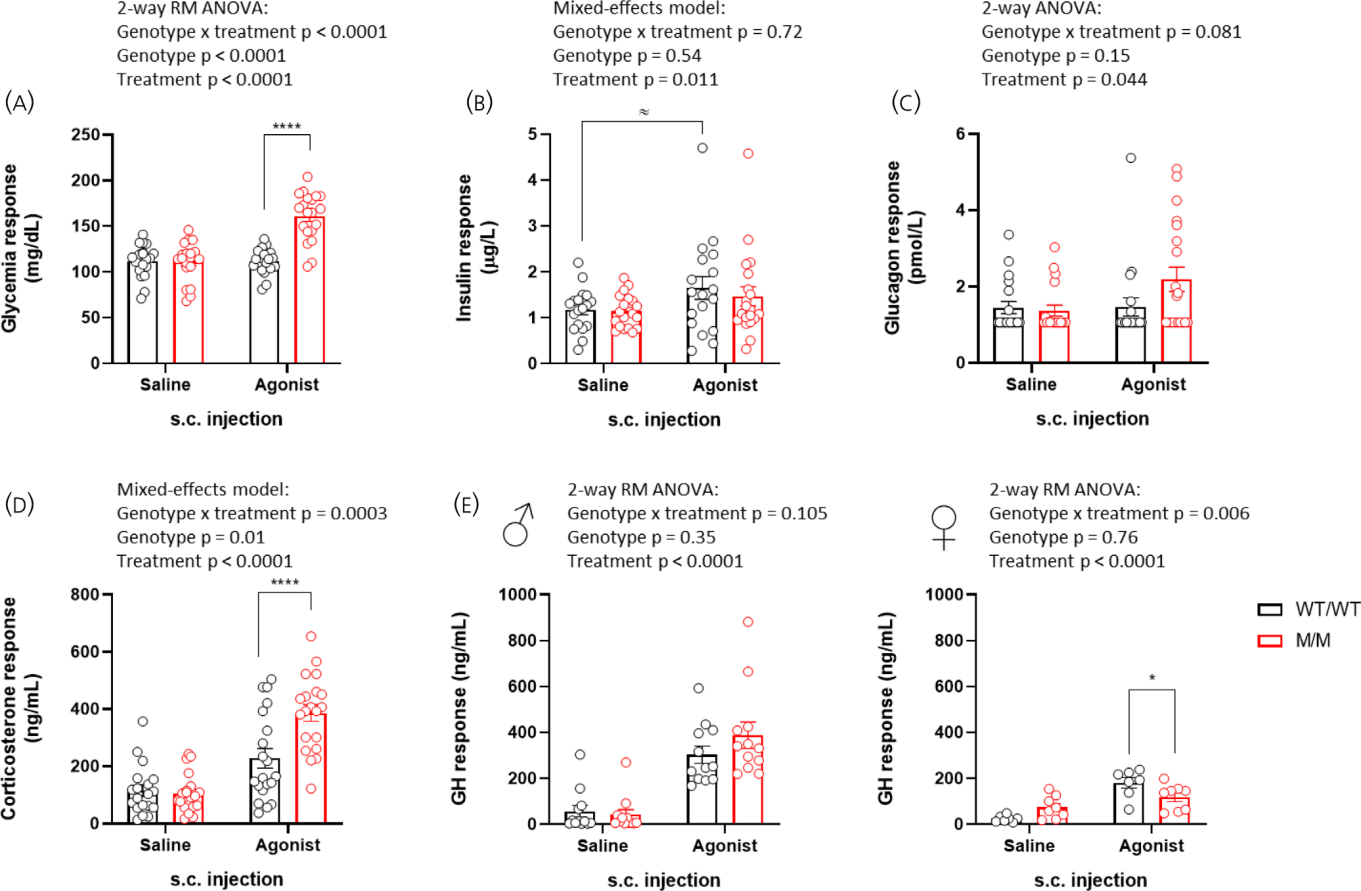
Early hormonal response to GHSR stimulation in M/M and WT/WT littermate rats. 15‐min response to female (*n* = 16) and male (*n* = 24) adult WT/WT and M/M rats (*n* = 20 by genotype) s.c. injected with a GHSR agonist (AEZS130, 3E^−6^ mol/kg) or saline according to a random crossover design protocol. Shown are (A) blood glucose, (B) insulin, (C) glucagon, (D) corticosterone, and (E) GH responses for male and female rats (left and right panel, respectively). Data are presented as mean ± SEM and were analyzed using 2‐way repeated‐measures ANOVA and Sidak's post hoc tests when relevant. ^≈^ *p* < .1, *p < .05, **p < .01, ***p < .001, ****p < .0001. All specific statistical information is reported in Table S1.

To elucidate the mechanisms underlying the clear blood glucose increase and the prominent hypolocomotor response observed in M/M rats following GHSR agonist administration, we hypothesized that the CRR might be differentially involved compared to WT/WT littermate controls. To test that, we measured several hormones in blood of WT/WT and M/M rats in the early phase of the response to a GHSR agonist or saline. As expected, the GHSR agonist enhanced blood glucose only in M/M rats (Figure 6A). Concurrently, this treatment tended to increase insulin levels in WT/WT rats but not in M/M rats (Figure 6B). Despite numerous values falling below detection limit in the glucagon assay, the p‐value approached statistical significance, suggesting a potentially increased glucagon response in agonist‐treated M/M rats (Figure 6C). Figure 6D illustrates a clear corticosterone response to GHSR agonism, which was higher in M/M rats compared to WT/WT controls (386 vs. 228 ng/mL, respectively), with no differences observed under saline conditions. Finally, as anticipated, GHSR agonist treatment elicited a clear GH response over saline treatment in WT/WT rats, independent of sex (Figure 6E). However, M/M rats showed a clear response in male rats that did not differ from their WT/WT counterparts. Unexpectedly, the response in female M/M rats appeared blunted. Collectively, these results suggest that corticosterone, and possibly glucagon, exhibit an enhanced response to GHSR stimulation in M/M rats compared to WT/WT littermate controls as explored in the early response to stimulation.

### 3.7 The GHSR‐Q343X isoform exhibits preserved Gα protein signaling repertoire but prolonged activation

**FIGURE 7:**
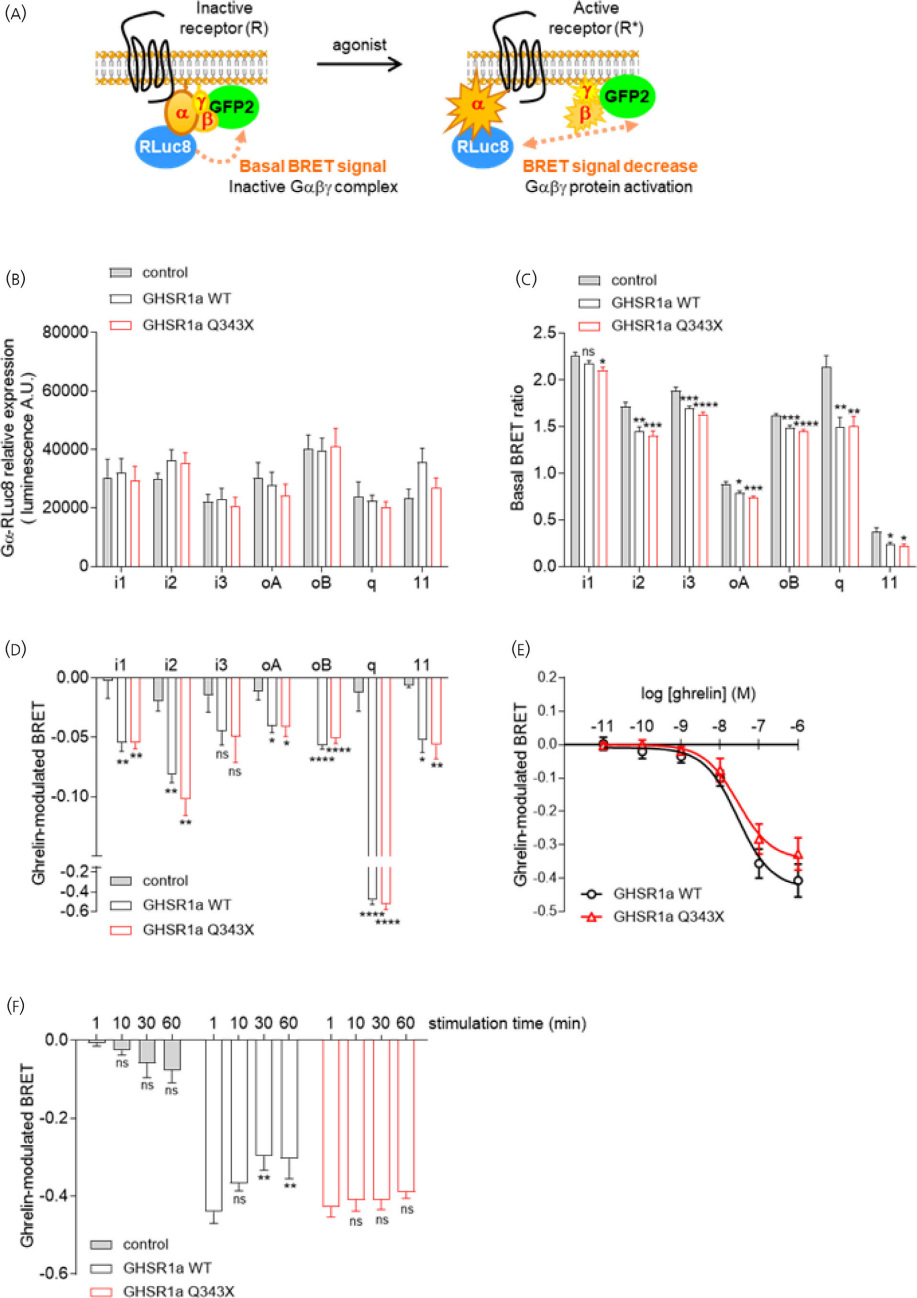
GHSR‐Q343X exhibits similar G protein signaling but prolonged activation compared to GHSR‐WT receptor. Schematic representation illustrating the BRET‐based G protein probes (A). Under basal conditions, the BRET signal arises from energy transfer between the energy donor RLuc8 fused to the Gα protein and the energy acceptor GFP2 fused to the Gγ2 protein and is indicative of the inactive Gαβ1γ2 complex. Activation of receptors by agonists or constitutive activation leads to G protein activation and subsequent dissociation, resulting in a reduction of the BRET signal. Relative expression of Gα‐RLuc8 protein probes assessed by luminescence measurement in HEK293T cells co‐expressing Gα‐RLuc8, GFP2‐Gγ2 and Gβ1 in the absence (control) or in the presence of GHSR WT or Q343X (B). Basal Gα protein activation evaluated by measuring basal BRET signal in HEK293T cells co‐expressing Gα‐RLuc8, GFP2‐Gγ2 and Gβ1 in the absence (control) or presence of GHSR WT or Q343X (C). Gα protein activation evaluated by measuring BRET signal in HEK293T cells co‐expressing Gα‐RLuc8, GFP2‐Gγ2 and Gβ1 in the absence (control) or in the presence of GHSR WT or Q343X, after stimulation with ghrelin (10^−6^ M) for 1 min (D). Dose–response curve performed in HEK293T cells co‐expressing Gαq‐RLuc8, GFP2‐Gγ2 and Gβ1 in the presence of GHSR WT or Q343X after stimulation with increasing concentrations of ghrelin (ranging from 10^−11^ to 10^−6^ M) for 1 min (E). Gαq protein activation was evaluated by measuring the BRET signal in HEK293T cells co‐expressing Gαq‐RLuc8, GFP2‐Gγ2 and Gβ1 in the absence (control) or in the presence of GHSR WT or Q343X, after stimulation or not with ghrelin (10^−6^ M) for different time periods (F). Results are expressed as the difference in the BRET signal measured in the presence and in the absence of ghrelin. Data represent the mean ± SEM of five or six independent experiments. Statistical significance between cells expressing GHSR (WT or Q343X) or not (control) was assessed using one‐way ANOVA followed by Bonferroni's multiple comparison test or two‐way ANOVA followed by Dunnett's multiple comparison test (Panel F). Significance levels are denoted as follows: *p < .05; **p < .01; ***p < .001; ****p < .0001; ns, not statistically significant.

Given that GHSR stimulation revealed an enhanced propensity of *Ghsr* ^Q343X^ rats to develop both metabolic and behavioral responses, we aimed to elucidate the mechanistic basis of this mutation and its consequences on GHSR signaling. Since the GHSR‐Q343X variant has been reported to preferentially engage G protein signaling pathways,^21^ we assessed G protein activation in HEK293T cells transiently expressing either the WT or Q343X receptors. We used BRET^2^ (Bioluminescence Resonance Energy Transfer)‐based probes, previously validated^22^, ^26^ to quantitatively assess both ligand‐independent (constitutive) and ligand‐dependent G protein activation. This assay relies on non‐radiative energy transfer between a Renilla luciferase (RLuc8) donor fused to the Gα subunit and a GFP2 acceptor fused to the Gγ2 subunit (Figure 7A). Under basal conditions, when the receptor adopts an inactive conformation, the preassembled Gαβ1γ2 heterotrimer maintains close proximity between the Gα and Gγ2 subunits, generating a measurable basal BRET signal. Constitutive or agonist‐induced receptor activation shifts the receptor equilibrium toward an active state, thereby promoting dissociation of the Gα and Gγ2 subunits and resulting in a decrease in the BRET signal (Figure 7A). Constitutive activation of GHSR refers to its intrinsic ability to activate downstream signaling pathways in the absence of agonist stimulation. This basal activity is a defining functional feature of the receptor and is crucial for maintaining its physiological role, as mutations that selectively disrupt GHSR constitutive signaling cause familial short stature syndrome.^16^ Expression of GHSR‐WT in HEK293T cells consistently resulted in a decreased basal BRET signal measured between the various Gα subunits (except Gαi1) and Gγ2, compared with control cells (Figure 7B,C). This reduction indicates that GHSR‐WT promotes dissociation of G protein subunits, reflecting constitutive activation of both Gαi/o and Gαq proteins in the absence of ligand (Figure 7C), consistent with previous reports.^15^, ^16^, ^26^ At comparable Gα subunit expression levels (Figure 7B), the GHSR‐Q343X mutant triggered a similar decrease in basal BRET signal, demonstrating a comparable extent of basal G protein activation as the WT receptor (Figure 7C), and indicating that the Q343X mutation does not alter the receptor's constitutive activity. Upon ghrelin stimulation, both WT and Q343X receptors displayed a robust and similar decrease in the BRET signal, corresponding to comparable G protein dissociation and reflecting similar G protein activation (Figure 7D). Furthermore, the potency of ghrelin in promoting Gαq activation was not significantly different between GHSR‐WT and GHSR‐Q343X expressing cells (Figure 7E). However, kinetic analyses revealed that ghrelin‐induced Gαq protein activation was more sustained over time in GHSR‐Q343X‐expressing cells compared to those expressing GHSR‐WT (Figure 7F). Collectively, these in vitro results indicate that the GHSR‐Q343X triggers prolonged G protein signaling, which is compatible with the impaired receptor internalization previously shown for this variant.^21^

## 4 DISCUSSION

By using animals with genetically heightened GHSR sensitivity, we demonstrated that GHSR agonism alone could induce a robust blood glucose increase reminiscent of a status of severe energy deficit, aligning with our hypothesis. Concurrently, *Ghsr* ^Q343X^ rats exhibited unexpected delayed feeding and hypolocomotor responses. This study questions whether GHSR responsivity upon GHSR agonism could prioritize protective responses such as glucose counter‐regulatory responses over certain behavioral responses such as feeding.

The present study unveiled that *Ghsr* ^Q343X^ rats, without alterations in glucose or insulin tolerance, exhibited a clear glucose elevation to GHSR agonism, independently of their feeding state. In contrast, WT/WT littermate rats displayed a modest and variable glucose elevation to agonism that only emerged under specific metabolic contexts. This observation aligns with the enhanced GHSR sensitivity initially proposed for these mutant rats.^21^ Indeed, ghrelin has been shown to elevate blood glucose levels in humans, mice, and rats at least in the fasted condition,^27^, ^28^, ^29^ a property that is blunted in *Ghsr* null mice.^30^ The demonstration that GHSR plays a key protective role against glucopenia has required mouse models under life‐threatening conditions of chronic severe caloric restriction.^4^ Interestingly, among rare human *GHSR* mutations documented so far, mostly in the heterozygous state, one well‐documented clinical case supports the hypothesis that abnormal GHSR functioning could enhance vulnerability to hypoglycemia in humans.^31^ In particular, the clinical record of a sporadic case of a patient diagnosed with isolated GH deficiency in the context of compound heterozygosity for *GHSR* mutations—a null mutation (*GHSR* ^W2X^) and a partial loss‐of‐function mutation (*GHSR* ^R237W^)—was characterized by very unusual recurrent hypoglycemic attacks until adolescence. Using the *Ghsr* ^Q343X^ model, we found that GHSR agonism was sufficient in carriers of the mutant allele (whether heterozygous or homozygous) to promote an increase in blood glucose, an observation relevant to rats of both sexes and in both adolescents and adults. Supporting this, we found that corticosterone and glucagon—hormones capable of raising blood glucose levels—were higher in GHSR‐stimulated M/M rats compared to WT/WT control littermates, while the hypoglycemic hormone insulin did not significantly differ among groups. In contrast, the GH response to GHSR stimulation in M/M rats showed an unexpected sex dimorphism. Indeed, male M/M rats responded similarly to WT/WT controls, an observation in agreement with our former results obtained in male M/M rats of the FHH strain when stimulated by a maximal dose of ghrelin, despite an enhanced response to lower doses of ghrelin.^21^ In comparison, the blunted GH response of female M/M rats to GHSR agonism is unexpected and suggests that the contribution of GH release to the blood glucose elevation, a feature shared by both sexes, may not be critical in our model. Overall, we found that the hormonal responses to GHSR stimulation in M/M rats, globally consistent with the known stimulatory properties of ghrelin on glucagon, corticosterone, or GH,^30^, ^32^, ^33^ could account to various degrees for the increased blood glucose response found in GHSR‐stimulated M/M rats. More broadly, these mediators, known components of the CRR, support the hypothesis of a relationship between ghrelin and CRR, as recently proposed. Precisely, *Ghrl* ^−/−^ mice, compared to wild‐type mice, showed a more pronounced hypoglycemia in response to acute insulin, a higher glucose infusion rate, and less robust corticosterone and GH responses during hyperinsulinemic–hypoglycemic clamps, effects that can be countered by GHSR agonism.^11^ Overall, using the *Ghsr* ^Q343X^ model, we propose that GHSR responsivity by itself could amplify the CRR, providing a novel perspective on the role of ghrelin in glycemic control.

Intriguingly, our dose–response curves of GHSR agonist have revealed that *Ghsr* ^Q343X^ carriers showed a distinctive hypolocomotor response occurring within minutes post‐injection and lasting 30–60 min. Additionally, the GHSR agonist was able to inhibit the rearing response associated with cocaine‐induced hyperlocomotion in M/M rats. Interestingly, this behavioral inhibition and the blood glucose increase observed in stimulated *Ghsr* ^Q343X^ rats exhibited a similar time course and reached a maximal response at a comparable dose of the GHSR agonist (1E^−6^ mol/kg, s.c.). Concurrently, the release of corticosterone following GHSR agonist treatment, compared to saline, was more pronounced in M/M rats than in WT/WT rats (3.7 fold vs. 1.9 fold, respectively). This observation aligns with the established role of GHSR in the stress response.^34^ However, the corticosterone response to acute stress did not differ across genotypes, as evidenced by a similar response to saline injection (Figure 6D) or contention stress (unpublished data in FHH rats). To account for the behavioral impairment found in stimulated M/M rats, a specific effect on locomotion is plausible. In particular, genetic and pharmacological studies have already established a role for GHSR in several mechanisms related to locomotor activity, including its role in dopaminergic neuro‐modulation and its involvement in food‐anticipatory activity and exercise endurance.^24^, ^35^, ^36^, ^37^ Yet, M/M rats, similar to WT/WT littermates, showed preserved food‐anticipatory activity to scheduled feeding^38^ and also preserved psychomotor sensitization to iterative injections of cocaine, as shown herein. Overall, these findings illustrate the feasibility for GHSR agonism to mediate a prominent hypolocomotor response and that *Ghsr* ^Q343X^ rats are more susceptible to these effects. Further investigations will be required to decipher the neuronal substrates behind this response.

Unlike other hormones, the gut hormone ghrelin shows orexigenic properties. Indeed, most models document a clear feeding response, as early as 1‐h post‐injection to several hours, following peripheral injections of ghrelin or GHSR agonists.^39^, ^40^, ^41^ Consistent with our previous study revealing a left‐shifted dose–response to a GHSR agonist (hexarelin) in M/M male rats of the FHH strain,^21^ an observation potentially explained by heightened GHSR sensitivity, M/M rats of the Wistar strain showed a significantly increased 4‐h feeding response to a lower dose of agonist over saline treatment compared to WT/WT control rats. However, as evidenced herein, *Ghsr* ^Q343X^ rats exhibited an unexpectedly altered feeding pattern in response to GHSR agonism, which can be divided into two periods. First, at an agonist dose that typically triggered a rapid feeding response within 30 min or 1 h post‐injection in WT/WT rats, the feeding response of M/M rats did not significantly differ from saline conditions; however, their locomotion was severely decreased. Second, at an agonist dose injected able to prolong the feeding response during the 2nd–4th hour interval post‐treatment in WT/WT rats, M/M rats showed a catch‐up feeding response that exceeded that of WT/WT rats, without any locomotor impairment. Overall, these findings suggest that enhancing GHSR sensitivity at the level of the receptor may dramatically alter the feeding response kinetics to GHSR agonism, even as the overall magnitude of the 4‐h feeding response remains conserved. To explain this unexpected feature, alterations in the feeding circuits of *Ghsr* ^Q343X^ rats seem unlikely, as evidenced by the potent catch‐up feeding once the hypolocomotor response lessened. Instead, we favor the hypothesis of a competition between on one side the synchronized glucose increase and behavioral inhibition, and on the other side the feeding response typically initiated by the injection and lasting several hours. This model therefore illustrates a paradoxical GHSR response that contrasts with the well‐established orexigenic effects of ghrelin. Such pharmacological response in *Ghsr* ^Q343X^ rats would suggest that globally enhancing GHSR sensitivity might prioritize one hormonal effect over another.

Based on the distinctive features of GHSR‐stimulated *Ghsr* ^Q343X^ rats, as identified in this study, we sought to improve our understanding of the mechanism of action of the GHSR‐Q343X mutation. To date, the only functional study dedicated to this mutation revealed that GHSR‐Q343X expressing cells enhanced GHSR‐G protein signaling over GHSR‐β‐arrestin signaling in cellular systems,^21^ although several signaling features remained unexplored. In our present work, we first observed that M/M and WT/WT rats showed similar neurocircuit sensitization to cocaine, a process recently shown to require functional GHSR‐β‐arrestin signaling.^25^, ^42^ Second, using specific BRET assays, we found that the GHSR‐G‐protein signaling repertoire was similar in cells expressing GHSR‐Q343X and GHSR‐WT, both in ghrelin‐dependent or ghrelin‐independent signaling contexts. Most notably, we disclosed that cells expressing GHSR‐Q343X displayed a maximal GHSR Gα protein response to ghrelin that was maintained over a longer timeframe compared to cells expressing GHSR‐WT. This observation is consistent with the previously documented altered GHSR internalization process.^21^ Overall, these findings further support a gain‐of‐function mechanism for the *Ghsr* ^Q343X^ allele, characterized by sustained signaling through the GHSR‐G protein pathway in response to ghrelin, while the GHSR‐β‐arrestin pathway appears unaltered. Furthermore, this mechanism aligns with the dominant mode of transmission of the *Ghsr* ^Q343X^ allele, as heterozygous rats exhibit the aforementioned distinctive pattern of response to GHSR agonism (not shown).

Physiological studies using the *Ghsr* ^Q343X^ model provided comprehensive insights into its features when raised under fed conditions without stress. Indeed, *Ghsr* ^Q343X^ rats, whether from the poly‐pathological FHH strain or the healthy Wistar strain (present study), exhibit a moderate phenotype, largely consistent with the chronic effects of ghrelin. In particular, these M/M rats showed a shift in nutrient partitioning toward carbohydrates in adulthood^38^ and increased body weight gain as fat and decreased insulin sensitivity as they age.^21^ Additionally, as shown herein, M/M rats gained slightly more weight during infancy compared to their WT/WT littermates, a modest effect potentially involving the enhanced effects of the GHSR on somatic growth. As disclosed herein, our finding reveals that GHSR‐stimulated *Ghsr* ^Q343X^ rats showed a remarkable pattern of response, thereby establishing a functional link between the GHSR and key ghrelin functions. This finding marks a significant advancement in the understanding of this genetic model, which was initially considered in some studies as a *Ghsr* hypo‐functioning model. Importantly, the blood glucose increase observed in M/M rats upon GHSR agonism elucidates why these rats maintained better blood glucose compared to their littermate controls when subjected to a chronic nutritional stress (calorie restriction).^38^ In other prospects, the enhanced corticosterone response found in GHSR‐stimulated M/M rats has plausibly biased earlier studies in their interpretation of GHSR behavioral responses. Specifically, similar to the acutely blunted feeding response found in GHSR‐stimulated Wistar‐*Ghsr* ^Q343X^ rats, FHH‐*Ghsr* ^Q343X^ rats injected with doses able to induce significant 1‐h food intake in littermate controls exhibited a blunted 1‐h feeding response to ghrelin^43^ or to the GHSR agonist hexarelin (our unpublished data). More globally, by examining the early response to GHSR agonism in *Ghsr* ^Q343X^ rats, we unveiled the versatile role of GHSR signaling. This model serves as a proof‐of‐concept, demonstrating the feasibility in animals with globally heightened GHSR responsivity to shift the orexigenic response to ghrelin agonism into an unexpected hypolocomotor response. This observation aligns with the newly assigned role of ghrelin in survival in response to exposure to chronic stressors (nutritional, social, physical or addiction drugs).^2^, ^34^, ^44^, ^45^ Based on our findings, we speculate that modifications in the threshold for ghrelin‐induced GHSR signaling in relevant cells could be part of the adaptive ghrelin response to chronic stress.

Given the distinctive pharmacological responses modeled in GHSR‐stimulated *Ghsr* ^Q343X^ rats, we then asked about their relevance in human pathophysiological conditions. While the lack of practical methods to measure central GHSR sensitivity in humans renders this consideration speculative, it is plausible that human conditions analogous to life‐threatening calorie restriction protocols used in mice to establish ghrelin's role in survival could be relevant.^2^, ^3^, ^4^, ^5^ Anorexia nervosa (AN) is a notable example of severe undernutrition characterized by critically low body weight and the inability to gain or maintain weight. In AN patients, elevated blood levels of ghrelin have been consistently documented,^46^, ^47^ leading to much debate regarding the apparent inefficacy of orexigenic ghrelin in improving the disease.^48^, ^49^ Based on the novel insights into GHSR functioning revealed by the *Ghsr* ^Q343X^ model, we propose that AN patients, during phases of severe undernutrition, may be more susceptible to the protective effects of ghrelin. These effects may include both beneficial glucoregulatory impacts and detrimental behavioral consequences.

Several limitations can be raised in the present study. First, the clearcut pattern of response to GHSR agonism found in *Ghsr* ^Q343X^ rats (i.e., blood glucose increase and hypolocomotor response), although likely relevant to pharmacotherapies against the GHSR, may not reflect a physiological setting. Second, while few reports document blood glucose elevation upon GHSR agonism in the rat species,^29^ it was intriguing that in contrast to M/M rats, WT/WT rats did not show a significant blood glucose increase in response to agonism in most conditions tested. Nevertheless, when we subjected rats to fluctuations of food availability, stimulated WT/WT rats showed a weak but significant increase in blood glucose, suggesting that the nutritional status is important for this property in rats. Third, the unexpected alteration of locomotion and feeding behavior of *Ghsr* ^Q343X^ rats in response to GHSR agonism remains unexplained. The hypothesis of a pharmacologically induced aversion induced by GHSR agonism in *Ghsr* ^Q343X^ rats cannot be formally excluded.

## 5 CONCLUSION

In a model of genetically heightened GHSR sensitivity, GHSR agonist stimulation was sufficient to promote a robust blood glucose increase, while the acute feeding response was delayed in a context of unexpected hypolocomotor response. This mechanism may have implications for severe states of undernutrition such as restrictive anorexia nervosa.

## AUTHOR CONTRIBUTIONS

**Philippe Zizzari**: Conceptualization; formal analysis; investigation; validation; writing—review and editing. **Olalla Ramírez‐Penas**: Investigation. **Véronique Pons**: Formal analysis; investigation; validation; writing—review and editing. **Matilda Oujezdska**: Investigation. **Ange‐Louis Sammarcelli**: Investigation. **Thomas Saint‐Yves**: Investigation. **Inès Chevallier‐Chantepie**: Investigation. **Lucile Baussart**: Investigation. **Vanessa Nhan**: Investigation. **Adèle Phalip**: Investigation. **Céline Gales**: Funding acquisition; resources; writing—review and editing. **Daniela Cota**: Funding acquisition; resources; writing—review and editing. **Florence Noble**: Funding acquisition; resources; writing—review and editing. **Jacques Pantel**: Conceptualization; formal analysis; investigation; project administration; resources; supervision; writing—original draft.

## CONFLICT OF INTEREST STATEMENT

The authors declare no conflicts of interest.

## Supporting information


**Table S1.** Summary of statistical analyses in rat studies.

## Data Availability

The data that support the findings of this study are available from the corresponding author upon reasonable request.
