# Supplementary material for: GHSR agonism increases blood glucose but delays food intake in GHSR hyperresponsive rats
Source: J Neuroendocrinol. 2026 Feb 13;38(2):e70143. doi: 10.1111/jne.70143 (PMC12905516; doi:10.1111/jne.70143)
Supplement: Supplementary file 1 — Table S1. Summary of statistical analyses in rat studies. [file JNE-38-e70143-s001.pdf]

Table S1. Summary of statistical analyses in rat studies.											
Figure	Test		sample size (n)	p value	Post hoc test	p value					
1A left panel, glucose response to agonist											
Male rats	Two-way RM ANOVA		WT/WT n= 8; M/M n= 8								
	Interaction	F (4, 56) = 14,83		P<0,0001							
	Time	F (2,562, 35,86) = 25,74		P<0,0001							
	Genotype	F (1, 14) = 9,565		P=0,0079							
Female rats	Two-way RM ANOVA		WT/WT n= 8; M/M n= 8								
	Interaction	F (4, 56) = 14,91		P<0,0001							
	Time	F (2,224, 31,14) = 37,55		P<0,0001							
	Genotype	F (1, 14) = 38,53		P<0,0001							
1A right panel											
	Two-way ANOVA				Sidak's multiple comparison test						
	Interaction	F (1, 28) = 3,661		P=0,0660	Males WT vs. M	0,0009					
	Sex	F (1, 28) = 9,287		P=0,0050	Females WT vs. M	<0,0001					
	Genotype	F (1, 28) = 56,89		P<0,0001							
1B, glycemia to agonist dose-response	Two-way RM ANOVA		WT/WT n= 6; M/M n= 6		Sidak's multiple comparison test						
	Interaction	F (4, 44) = 18,76		P<0,0001	WT vs. M, saline	0,9979					
	Dose	F (3,220, 35,42) = 7,898		P=0,0003	WT vs. M, AEZS-130 1E-7M	0,9108					
	Genotype	F (1, 11) = 12,85		P=0,0043	WT vs. M, AEZS-130 3E-7M	0,2268					
					WT vs. M, AEZS-130 1E-6M	0,0014					
					WT vs. M, AEZS-130 3E-6M	0,0011					
1C left panel, glucose response to ghrelin	Two-way RM ANOVA		WT/WT n= 6; M/M n= 6		Sidak's multiple comparison test						
	Interaction	F (3, 30) = 4,800		P=0,0076	WT vs. M, t0	0,7669					
	Time	F (3, 30) = 12,01		P<0,0001	WT vs. M, t15	0,0117					
	Genotype	F (1, 10) = 9,501		P=0,0116	WT vs. M, t30	0,0135					
					WT vs. M, t60	0,0932					
1C right panel, AOC	Two-tailed unpaired t test	t=3,856, df=10		0,0032							
1D, Body weight response to feeding fluctuations	Two-way RM ANOVA		WT/WT n= 12; M/M n= 12								
	Interaction	F (9, 198) = 0,07649		P=0,9999							
	Days	F (9, 198) = 183,4		P<0,0001							
	Genotype	F (1, 22) = 0,07420		P=0,7879							
1E, Glucose response to feeding fluctuations	Three-way RM ANOVA										
	Treatment	F(1,22) = 110,92		P<0,001							
	Nutritional status	F(5,110) = 71,06		P<0,001							
	Genotype	F(1,22) = 40,01		P<0,001							
	Genotype * Treatment	F(1,22) = 47,01		P<0,001							
	Nutritional status * Treatment	F(5,110) = 3,42		P=0,006							
	Genotype * Nutritional status* Treatment	F(5,110) = 4,79		P=0,0005							
	Global effects										
	Genotype	Mean ± SE (mg/dL)	95% Cl	erence (t15	t-value	p-value					
	WT, t0	114.0 ± 2.84	[108.1, 119.9]	+7.39	t(22) = -2.599	0.0164					
	WT, t15	121.4 ± 2.84	[116.1, 127.9]								
	MM, t0	118.0 ± 2.84	[112.1, 123.9]	+34.96	t(22) = -12.296	< 0.0001					
	MM, t15	152.9 ± 2.84	[147.1, 158.9]								
	**Nutritional Status**	**Estimate**	**SE**	**df**	**t.ratio**	**p-value**					
	4d-CR (d16), WT, t0-t15	-10.67	4.61	99.3	-2.315	0.0227					
	4d-CR (d16), M, t0-t15	-23.50	4.61	99.3	-5.100	<0.0001					
	Adlib (d1), WT,t0-t15	-3.00	4.61	99.3	-0.651	0.5165					
	Adlib (d1), M, t0-t15	-51.00	4.61	99.3	-11.067	<0.0001					
	Fast (d2), WT, t0-t15	-8.42	4.61	99.3	-1.826	0.0708					
	Fast (d2), M, t0-t15	-29.75	4.61	99.3	-6.456	<0.0001					
	Fed (d7), WT, t0-t15	-9.50	4.61	99.3	-2.062	0.0419					
	Fed (d7), M, t0-t15	-43.00	4.61	99.3	-9.331	<0.0001					
	Fed (d8), WT, t0-t15	-9.00	4.61	99.3	-1.953	0.0536					
	Fed (d8), M, t0-t15	-37.92	4.61	99.3	-8.228	<0.0001					
	Refed (d3), WT, t0-t15	-3.75	4.61	99.3	-0.814	0.4177					
	Refed (d3), M, t0-t15	-24.58	4.61	99.3	-5.335	<0.0001					
2A, Body weight	General Mixed Model Information		WT/WT n= 215; M/M n= 230								
	Criterion	Model 1	Model 2								
	Formula	Weight ~ Time * Genotype * Sex + (1 | Litter) + (1 | ID)	Weight ~ Time * Genotype * Sex + (Time | Litter) + (1 | ID)								
	Fitting Method	REML, t-tests (Satterthwaite)	REML, t-tests (Satterthwaite)								
	REML Criterion	7689.5	7629.7								
	Scaled Residuals	Min: -4.22, Q1: -0.42, Median: -0.01, Q3: 0.38, Max: 4.18	Min: -4.22, Q1: -0.42, Median: -0.02, Q3: 0.39, Max: 3.42								
	Number of Observations	890	890								
	Groups (ID; Litter)	445; 14	445; 14								
	General Mixed Model Information										
	Group	Effect	Variance (Model 1)	Std.Dev (Model 1)	Variance (Model 2)	Std.Dev (Model 2)	Correlation (Model 2)				
	ID	(Intercept)	64.58	8.04	86.63	9.31	-				
	Litter	(Intercept)	82.80	9.10	64.14	8.01	-				
	Litter	Time	-	-	5.32	2.31	-0.68				
	Residual	-	267.35	16.35	222.86	14.93	-				
	General Mixed Model Information										
	Effect	Estimate (Model 1)	Std. Error (Model 1	f (Model 1)	t value (Model 1)	e (Model 1)	nate (Model 2)	Error (Model 2)	df (Model 2)	alue (Model 2)	alue (Model 2)
	(Intercept)	-40.03	4.02	74.97	-9.95	< 0.001	-39.62	3.79	49.65	-10.44	< 0.001
	Time	32.71	0.50	441.00	65.97	< 0.001	32.67	0.79	24.72	41.35	< 0.001
	Genotype (WT/WT)	5.28	4.40	687.84	1.20	0.230	6.02	4.12	707.59	1.46	0.144
	Sex (M)	-58.91	4.12	722.16	-14.29	< 0.001	-59.69	3.99	642.86	-14.97	< 0.001
	Time:Genotype (WT/WT)	-1.70	0.70	441.00	-2.43	0.015	-1.84	0.64	429.14	-2.87	0.004
	Time:Sex (M)	20.56	0.64	441.00	32.00	< 0.001	20.67	0.63	438.91	32.97	< 0.001
	Genotype (WT/WT):Sex (M)	3.86	5.65	688.09	0.68	0.495	2.79	5.29	708.03	0.53	0.598
	Time:Genotype (WT/WT):Sex (M)	-1.62	0.90	441.00	-1.81	0.072	-1.42	0.82	429.37	-1.72	0.086
2B, left panel OGTT	Two-way RM ANOVA		WT/WT n= 13 ; M/M n= 11								
	Interaction	F (4, 44) = 0,9918		P=0,4220							
	Time	F (1,300, 14,30) = 11,39		P=0,0027							
	Genotype	F (1, 11) = 1,304		P=0,2778							
2B, right panel OGTT	Two-way ANOVA				Sidak's multiple comparison test						
	Interaction	F (1, 20) = 9,146		P=0,0067	WT vs. M, saline	0,6612					
	Pre treatment	F (1, 20) = 8,368		P=0,0090	WT vs. M, agonist	0,0064					
	Genotype	F (1, 20) = 3,629		P=0,0713							
2C, left panel ITT	Two-way RM ANOVA		WT/WT n= 12 ; M/M n= 12								
	Interaction	F (8, 176) = 0,7426		P=0,6538							
	Time	F (3,199, 70,39) = 61,71		P<0,0001							
	Genotype	F (1, 22) = 1,745		P=0,2001							
2C, center panel KITT	Two-tailed unpaired t test	t=0,03701, df=22		0,9708							
2C, right panel AOC	Two-tailed unpaired t test	t=0,7257, df=22		0,4757							
3A, Locomotor response	Two-way RM ANOVA		WT/WT n= 20 ; M/M n= 20		Sidak's multiple comparison test						
	Interaction	F (2, 74) = 11,14		P<0,0001	WT vs. M, saline	0,9813					
	TTT	F (1,490, 55,14) = 39,67		P<0,0001	WT vs. M, agonist 1E-6mol/Kg	0,0002					
	Genotype	F (1, 37) = 20,80		P<0,0001	WT vs. M, agonist 3E-6mol/Kg	<0,0001					
3B, Feeding response	Two-way RM ANOVA				Sidak's multiple comparison test						
	Interaction	F (2, 58) = 7,033		P=0,0018	WT vs. M, saline	0,9945					
	TTT	F (1,700, 49,31) = 4,694		P=0,0179	WT vs. M, agonist 1E-6mol/Kg	0,2345					
	Genotype	F (1, 29) = 6,373		P=0,0173	WT vs. M, agonist 3E-6mol/Kg	0,0065					
4B, 4h Food intake	Two-way RM ANOVA		WT/WT n=32 ; M/M n=32		Sidak's multiple comparison test						
	Interaction			P=0.0053	WT vs. M, agonist 1E-8mol/Kg	0,0164					
	Dose			P<0.0001	WT, saline vs. agonist 1E-6mol/Kg	<0,0001					
	Genotype			P=0.6134	M, saline vs. agonist 1E-7mol/Kg	0,0477					
					M, saline vs. agonist 1E-6mol/Kg	<0,0001					
4C, 4h Locomotor response	Two-way RM ANOVA				Sidak's multiple comparison test						
	Interaction			P=0.0002	WT vs. M, agonist 1E-6mol/Kg	<0.0001					
	Dose			P<0.0001							
	Genotype			P=0.0103							
4D, Food intake	Two-way RM ANOVA				Sidak's multiple comparison test						
	Interaction	F (1, 62) = 7,724		P=0,0072	WT vs. M, interval 0-1h	0,3111					
	Time	F (1, 62) = 0,09563		P=0,7582	WT vs. M, interval 2-4h	0,0055					
	Genotype	F (1, 62) = 1,938		P=0,1689							
4E, Locomotor response	Two-way RM ANOVA				Sidak's multiple comparison test						
	Interaction	F (1, 61) = 39,97		P<0,0001	WT vs. M, interval 0-1h	<0,0001					
	Time	F (1, 61) = 0,008852		P=0,9254	WT vs. M, interval 2-4h	0,8243					
	Genotype	F (1, 61) = 29,90		P<0,0001							
5A left panel, Novelty test	Two-way RM ANOVA		WT/WT n=36 ; M/M n=30								
	Interaction	F (11, 726) = 2,310		P=0,0087							
	Time	F (6,303, 416,0) = 164,8		P<0,0001							
	Genotype	F (1, 66) = 1,254		P=0,2669							
5A right panel, Novelty test	Two-tailed unpaired t test	t=2,305, df=64		0,0244							
5B right panel, cocaine sensitisation	Two-way RM ANOVA				Sidak's multiple comparison test						
	Interaction	F (6, 360) = 0,5748		P=0,7504	WT, Cocaine d3 vs. Cocaine d5	0,0093					
	Injection	F (4,835, 290,1) = 54,25		P<0,0001	WT, Cocaine d3 vs. Cocaine d6	0,0033					
	Genotype	F (1, 60) = 0,7474		P=0,3908	WT, Cocaine d3 vs. Cocaine d7	0,0012					
					M, Cocaine d3 vs. Cocaine d5	0,003					
					M, Cocaine d3 vs. Cocaine d6	0,0037					
					M, Cocaine d3 vs. Cocaine d7	0,0063					
5C right panel, horizontal activity response	Two-way ANOVA				Sidak's multiple comparison test						
	Interaction	F (2, 90) = 1,664		P=0,1952	WT vs. M, sal/sal	0,9991					
	Treatment	F (2, 90) = 27,94		P<0,0001	WT vs. M, sal/cocaine	0,7207					
	Genotype	F (1, 90) = 4,494		P=0,0368	WT vs. M, ago/cocaine	0,0666					
5D right panel, rearing response	Two-way ANOVA				Sidak's multiple comparison test						
	Interaction	F (2, 88) = 7,750		P=0,0008	WT vs. M, sal/sal	>0,9999					
	Treatment	F (2, 88) = 10,09		P=0,0001	WT vs. M, sal/cocaine	0,994					
	Genotype	F (1, 88) = 7,374		P=0,0080	WT vs. M, ago/cocaine	<0,0001					
6A, Glycemia response	Two-way RM ANOVA		WT/WT n=20 ; M/M n=20		Sidak's multiple comparisons test						
	Interaction	F (1, 37) = 37,39		P<0,0001	WT vs. M, saline	0,9959					
	Genotype	F (1, 37) = 22,67		P<0,0001	WT vs. M, agonist 3E-6	<0,0001					
	Treatment	F (1, 37) = 35,99		P<0,0001							
6B, Insulin response	Mixed-effects model (REML)										
	Interaction	F (1, 33) = 0,1333		0,7174							
	Genotype	F (1, 37) = 0,3834		0,5396							
	Treatment	F (1, 33) = 7,158		0,0115							
6C, Glucagon response	Two-way ANOVA				Sidak's multiple comparisons test						
	Interaction	F (1, 74) = 3,124		P=0,0813	saline vs. Agonist 3E-6, WT	0,9758					
	Genotype	F (1, 74) = 2,101		P=0,1514	saline vs. Agonist 3E-6, M	0,0173					
	Treatment	F (1, 74) = 4,187		P=0,0443							
6D, Corticosterone response	Mixed-effects model (REML)				Sidak's multiple comparisons test						
	Interaction	F (1, 36) = 16,08		0,0003	WT vs. M, saline	0,9663					
	Genotype	F (1, 37) = 7,327		0,0102	WT vs. M, agonist 3E-6	<0,0001					
	Treatment	F (1, 36) = 75,99		<0,0001							
6E, GH response in male rats	Two-way RM ANOVA				Sidak's multiple comparisons test						
	Interaction	F (1, 23) = 2,848		P=0,1050	WT vs. M, saline	0,9635					
	Genotype	F (1, 23) = 0,8991		P=0,3529	WT vs. M, agonist 3E-6	0,1608					
	Treatment	F (1, 23) = 90,44		P<0,0001							
6F, GH response in female rats	Two-way RM ANOVA				Sidak's multiple comparisons test						
	Interaction	F (1, 13) = 10,43		P=0,0066	WT vs. M, saline	0,111					
	Genotype	F (1, 13) = 0,09324		P=0,7649	WT vs. M, agonist 3E-6	0,0436					
	Treatment	F (1, 13) = 33,57		P<0,0001							
